# 5q deletion in childhood T-acute lymphoblastic leukemia at diagnosis: a case report

**DOI:** 10.1186/s13256-024-04999-x

**Published:** 2025-06-12

**Authors:** Yousra Sbibih, Mohammed Bensalah, Mounia Slaoui, Abderrazak Saddari, Nabiha Trougouty, Abdelilah Berhili, Rachid Seddik

**Affiliations:** 1 Faculty of Medicine and Pharmacy, University Mohammed First , Oujda, Morocco; 2 Laboratory of Hematology , Mohammed VI University Hospital, Oujda, Morocco

**Keywords:** T-acute lymphoblastic leukemia, 5q deletion

## Abstract

**Background:**

We present the case of a 6-year-old Moroccan male patient of Berber ethnic origin, diagnosed with T-cell acute lymphoblastic leukemia, who exhibited a deletion of the 5q region.

**Case presentation:**

The patient initially presented with classic symptoms of T-cell acute lymphoblastic leukemia, including bone pain, hepatosplenomegaly, and lymphadenopathy. Laboratory tests revealed anemia, hyperleukocytosis, and a high percentage of lymphoid blasts in both the blood and bone marrow. Immunophenotyping results confirmed that these blasts were of T-cell origin. Cytogenetic analysis identified a deletion of the long arm of chromosome 5 in a subset of the patient’s cells.

**Conclusion:**

The presence of a 5q deletion in pediatric T-cell acute lymphoblastic leukemia is an unusual finding and its prognostic significance may differ from that observed in myeloid leukemias. The implications of this cytogenetic anomaly in lymphoid malignancies remain unclear and warrant further investigation. Understanding the origins and effects of such chromosomal abnormalities in T-cell acute lymphoblastic leukemia could provide deeper insights into the disease’s pathogenesis and contribute to more tailored therapeutic strategies.

## Introduction

The 5q− syndrome is a cytogenetic anomaly associated with myelodysplastic syndrome (MDS) and acute myeloid leukemia (AML) associated with myelodysplastic syndrome in adults and very rarely in children [1]. The occurrence of a deletion of the long arm of chromosome 5 in T-cell acute lymphoblastic leukemia (T-ALL) in children is an exceptional event. This anomaly is mainly associated with multiple cytogenetic abnormalities thought to be due to clonal expansion from an abnormal pluripotent stem cell. The profound heterogeneity of cytogenetic abnormalities found in acute T-cell lymphoblastic leukemia in the pediatric population is responsible for a wide variation in prognosis and therapeutic response. Hence, the interest in investigating these abnormalities to establish the best treatment for the patient, taking into account all associated risks. We report the clinical observation of a child diagnosed with T-ALL with 5q deletion.

## Case report

**Fig. 1:**
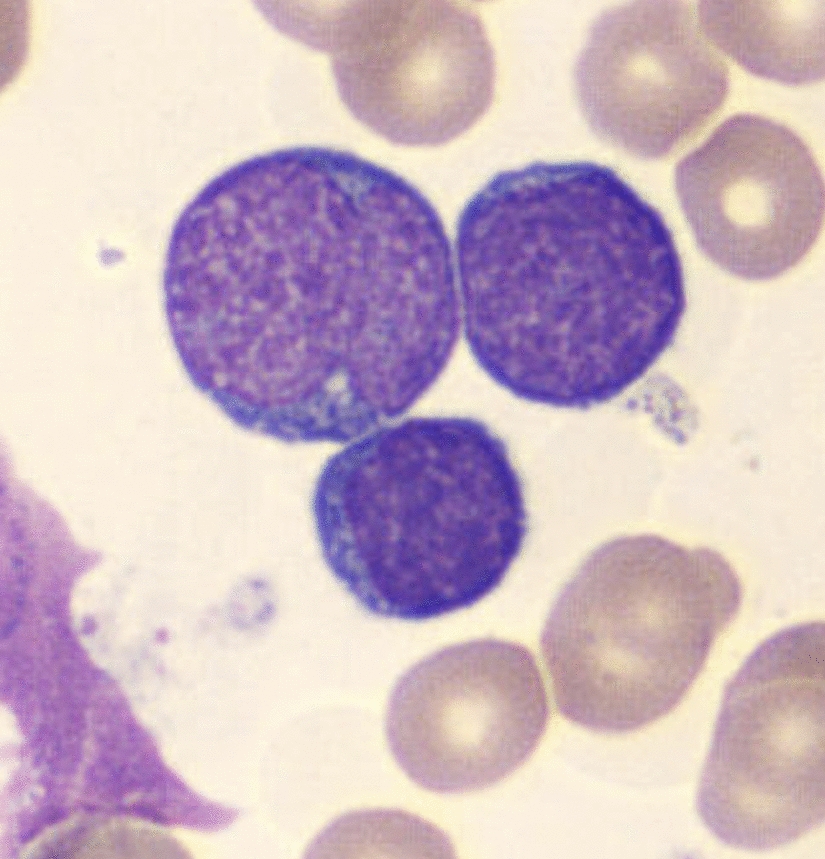
Blasts on peripheral blood smear

The case concerned a 6-year-old Moroccan male child, of Berber ethnic origin, KY, from a nonconsanguineous marriage, the third of four siblings, originally from Nador (a city located in the northeastern part of the country). Regarding the patient’s history, the pregnancy was well monitored and carried to term, with a vaginal delivery resulting in a birth weight of 3 kg. He was exclusively breastfed for 20 days, with diversification starting at 6 months. He received complete vaccination according to Morocco’s national immunization program. The patient has a surgical history of circumcision at the age of one, with no postoperative complications. The history of the illness dates back 3 months, beginning with the onset of pallor associated with significant asthenia, bone pain, and progressive weight loss that was overlooked by the family. The progression was marked by the onset of pain in the right upper quadrant, prompting the family to seek emergency care at the hospital. On physical examination, the child was afebrile and presented with generalized mucocutaneous pallor without purpuric or ecchymotic spots. He also presented with a tumor syndrome consisting of hepatosplenomegaly and bilateral cervical and inguinal adenopathies. Otorhinolaryngology examination revealed erythematopultaceous angina. The complete blood count (CBC) showed anemia with hemoglobin at 4.6 g/dL and hyperleukocytosis at 333 G/L. The blood smear showed 92% blasts (Fig. 1), 6% polynuclear neutrophils (PNN), and 2% lymphocytes. Platelet count was normal at 193 G/L.

**Fig. 2:**
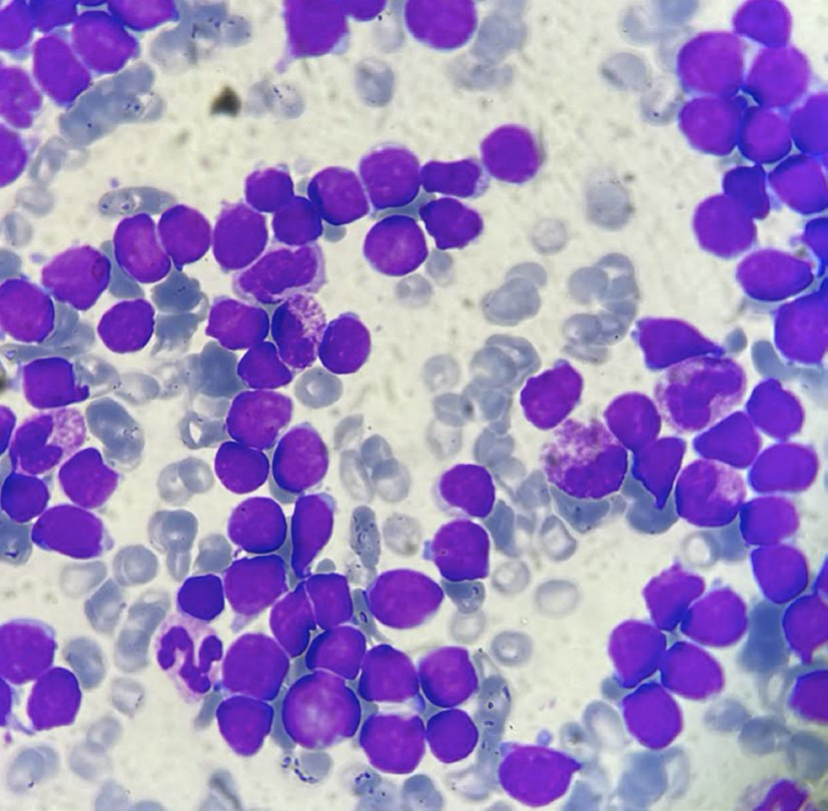
Blasts on bone marrow smear

**Fig. 3:**
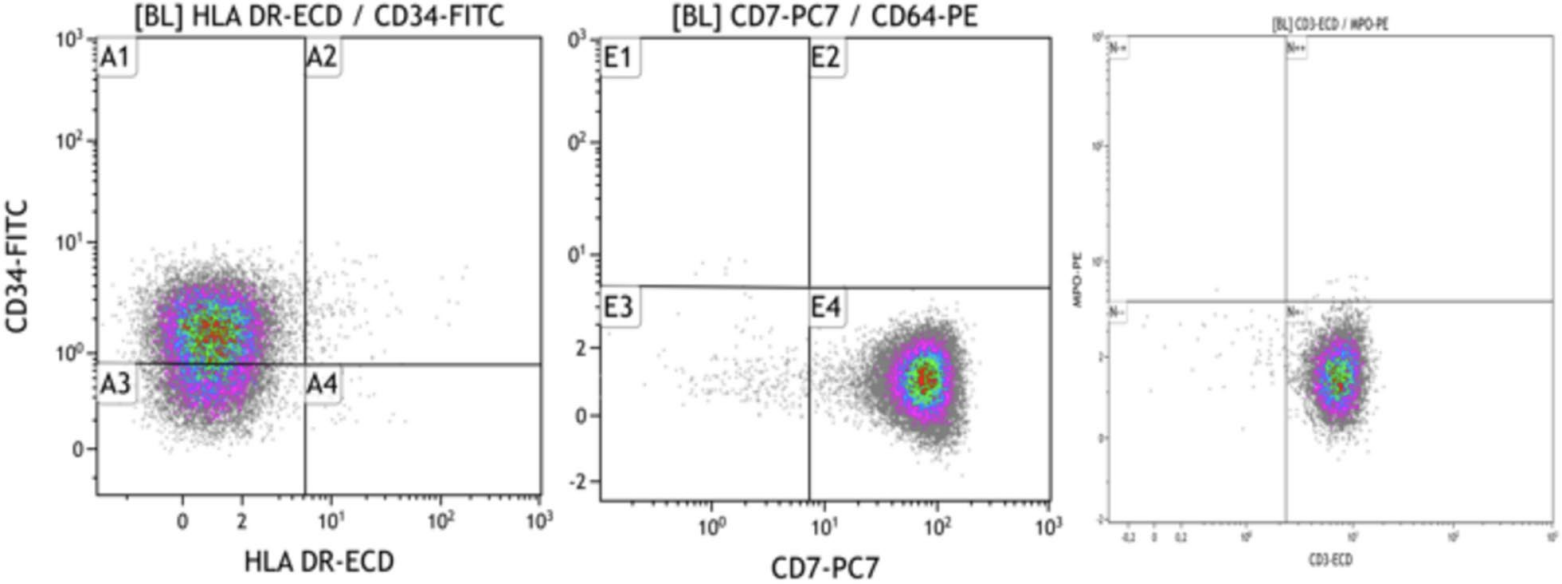
Immunophenotyping of bone marrow blasts

Bone marrow aspiration yielded a richly cellularized sample with 90% invasion by myeloperoxidase-negative lymphoid blasts (Fig. 2). Immunophenotyping of the bone marrow sample showed a 91% blastic population expressing the immaturity marker CD34, the lymphoid markers CD7, and CD3 intra (Fig. 3) and not expressing the HLA-DR markers CD33, CD117, CD13, MPO intra, CD64, CD79a, CD19, CD10, and CD20. In light of these findings, the diagnosis of acute T-cell lymphoblastic leukemia was made. A bone marrow karyotype was performed, and 4 out of 15 mitoses analyzed showed a pseudodiploid karyotype with a clonal structural anomaly—deletion of the long arm of chromosome 5.

The progression was marked by corticosteroid resistance and a blast percentage of 4% after the end of the induction phase and a rate of 3% at the end of consolidation. The patient is undergoing the MARALL chemotherapy protocol, which consists of a 7-week induction phase, followed by a 2-month consolidation phase, intensification phase one for 2 months, a 2-month interphase, intensification phase two for 2 months, and finally, maintenance treatment lasting 24 months. Clinically, we observed a significant improvement in the patient’s overall condition 5 months after the initiation of maintenance treatment.

## Discussion

Terminal or interstitial deletion of the long arms of chromosome 5 was initially described in 1974 by Van den Berghe *et al*. [2]. It has, since then, been described either as a sole rearrangement or in association with other chromosomal abnormalities in a wide variety of disorders of myeloid cell lineage, such as myelodysplasia [3–7], acute myeloid leukemia [7–11], polycythemia vera [3, 7], myelofibrosis [7–14], and essential thrombocythemia [14, 15]. A study published in 2016 by Roberta La Starza *et al*. carried out in collaboration with Belgian and Italian researchers in hemato-oncology, genetics, and molecular biology demonstrated deletion of the long arm of chromosome 5 in 23 out of 200 cases of T-ALL. Blasts showed early maturation arrest with expression of myeloid markers [16]. The particularity of our patient was that he presented this lesion at the time of diagnosis. Furthermore, our patient’s blasts did not express any myeloid markers. Deletions occurring on the long arm of chromosome 5 can lead to the loss of tumor suppressor genes, potentially influencing the development of certain diseases. This chromosome segment contains numerous genes crucial for blood cell formation (hematopoiesis), including transcription factors, cytokines, receptors, and cell cycle regulators. Notable genes in this region include RPS14 (critical for red blood cell development), a microRNA cluster (important for platelet-forming cells), and EGR1 (involved in the proliferation of blood stem cells). Although a specific tumor suppressor gene has not been pinpointed in acute lymphoblastic leukemia (ALL), several potential candidates have been identified, such as NR3C1 and TCF7, within the commonly deleted region at 5q31. Other genes, such as TRIM41, ZFP62, MAPK9, MGAT1, and CNOT6, located at 5q35, have been found to be down-regulated in T-cell ALL [16]. The varying size and patterns of deletions on 5q suggest that the loss of multiple genes, either individually or in combination, contributes to disease development. The absence of a single critical gene indicates that different combinations of deleted genes may result in a spectrum of clinical manifestations, ranging from myeloid malignancies to lymphoid leukemia [17]. Future research should focus on the interaction of deleted genes with the signaling pathways specific to T-ALL. Experimental and clinical studies are needed to explore the combined effects of deletions on the development of leukemic cells and their response to treatment. Molecular biology and omics techniques, such as genomics, transcriptomics, and proteomics, can play a crucial role in improving the management of patients with T-acute lymphoblastic leukemia (T-ALL). These approaches enable a comprehensive characterization of genetic abnormalities and gene expression profiles, thereby facilitating patient stratification according to risk and the personalization of treatments. For example, genomics can identify specific mutations and deletions, while transcriptomics can reveal expression signatures that predict responses to therapies. Additionally, proteomics can help us understand altered signaling pathways, paving the way for targeted therapies. By integrating these data, clinicians can develop more effective and tailored therapeutic strategies, thus improving clinical outcomes.

## Conclusion

Deletion of the long arm of chromosome 5 in the context of acute lymphocytic leukemia is a rare event. It may be interstitial or terminal. The literature review suggests that del(5q) is more frequent in children and does not represent a poor prognostic factor compared with its occurrence in myeloid leukemia. The discovery of this cytogenetic abnormality is almost always associated with the presence of myeloid markers on the blasts of acute lymphocytic leukemia. However, our patient showed no myeloid markers, which calls for further studies and research to understand the origin of the deletion on lymphoid blasts.

## Data Availability

We declare that we have all supporting data.
